# Certainty rating in pre-and post-tests of study modules in an online clinical pharmacy course - A pilot study to evaluate teaching and learning

**DOI:** 10.1186/s12909-016-0783-1

**Published:** 2016-10-14

**Authors:** Karen Luetsch, Judith Burrows

**Affiliations:** School of Pharmacy, The University of Queensland, 21 Cornwall St, Woolloongabba, Qld 4102 Australia

**Keywords:** Formative assessment, Formative feedback, e-learning, Certainty based marking, Pharmacy, Postgraduate

## Abstract

**Background:**

Graduate and post-graduate education for health professionals is increasingly delivered in an e-learning environment, where automated, continuous formative testing with integrated feedback can guide students’ self-assessment and learning. Asking students to rate the certainty they assign to the correctness of their answers to test questions can potentially provide deeper insights into the success of teaching, with test results informing course designers whether learning outcomes have been achieved. It may also have implications for decision making in clinical practice.

**Methods:**

A study of pre-and post-tests for five study modules was designed to evaluate the teaching and learning within a pharmacotherapeutic course in an online postgraduate clinical pharmacy program. Certainty based marking of multiple choice questions (MCQ) was adapted for formative pre- and post-study module testing by asking students to rate their certainty of correctness of MCQ answers. Paired t-tests and a coding scheme were used to analyse changes in answers and certainty between pre-and post-tests. A survey evaluated students’ experience with the novel formative testing design.

**Results:**

Twenty-nine pharmacists enrolled in the postgraduate program participated in the study. Overall 1315 matched pairs of MCQ answers and certainty ratings between pre- and post-module tests were available for evaluation. Most students identified correct answers in post-tests and increased their certainty compared to pre-tests. Evaluation of certainty ratings in addition to correctness of answers identified MCQs and topic areas for revision to course designers. A survey of students showed that assigning certainty ratings to their answers assisted in structuring and focusing their learning throughout online study modules, facilitating identification of areas of uncertainty and gaps in their clinical knowledge.

**Conclusions:**

Adding certainty ratings to MCQ answers seems to engage students with formative testing and feedback and focus their learning in a web-based postgraduate pharmacy course. It also offers deeper insight into the successful delivery of online course content, identifying areas for improvement of teaching and content delivery as well as test question design.

**Electronic supplementary material:**

The online version of this article (doi:10.1186/s12909-016-0783-1) contains supplementary material, which is available to authorized users.

## Background

Continuing professional development, graduate and postgraduate programs in health sciences and clinical education in Australia and many other countries are increasingly delivered online to accommodate the needs of adult, professional learners and address their expectations to be able to work, study and learn wherever and whenever they choose [1]. Evidence for comparable learning outcomes between online, internet-based and face-to-face course delivery has generally been established, although strategies for successful e-teaching and e-learning design and its effective implementation are still emerging [2–5]. A systematic review of internet based learning (IBL) in health profession education identified teaching strategies with a positive impact on learning outcomes, namely interactivity, practice exercises, repetition and feedback [6].

Formative assessment is regarded as essential in providing opportunities for learners to develop self-assessment and self-regulation skills [7, 8], optimise learning [9, 10] and prepare for summative assessment [11]. It offers guidance to adult, postgraduate e-learners who often study in relative isolation, asynchronous to others, in structuring their learning. Using assessment results to improve teaching practices and assessing the assessment can assist designers of e-learning for health professionals to meet the challenge of developing courses which are student-centred, relevant and applicable to learners who bring varying priorities to their course of study [12]. Continuous monitoring and evaluation of students’ results in formative tests allows for timely adjustment of learning content and delivery as well as assessment tasks to optimise student learning [13, 14].

One convenient strategy for formative assessment within the virtual learning environment (VLE) is the use of tests of multiple choice questions (MCQs) at the completion of learning modules [15]. MCQs have been validated as an assessment method in health sciences and clinical education, with diligent design contributing to test reliability and validity and the assessment of critical thinking [16–20]. The use of context-rich MCQs which test the application of clinical and therapeutic knowledge after educational activities promotes retention and application of knowledge [21].

The Postgraduate Clinical Pharmacy Programs (PCPP) at the University of Queensland (UQ), Australia, are delivered via a virtual learning platform, Blackboard® (Blackboard Inc., Washington DC, USA), and offer practicing pharmacists from Australia and other countries the opportunity to attain a postgraduate degree at a Diploma or Master’s level via course work. The program is structured into courses comprising of learning modules. These modules offer a wide range of learning content and activities to accommodate practicing pharmacists’ varying professional experience and background, scopes of practice and technological expertise. Module content is designed to build on pharmacists’ varying degrees of baseline clinical skills and knowledge, engaging them in critical thinking, reflection on their practice and discussing changes in clinical evidence and recent controversies. The program emphasises the teaching strategies delineated in Cook’s review [6], with formative assessment and feedback the focus of this evaluation.

Formative post-module MCQ have always been an integral component of the therapeutics online courses in the PCPP to encourage self-assessment of learning and prepare students for an open-book, computer-based, end of course MCQ exam [7]. The exam forms one aspect of summative assessment along with performance- and practice- based assessments [22].

Adding pre- module MCQ tests to post- tests integrates feeding forward and allows e-learners to self-regulate and focus their learning through the online study content, based on their pre-existing knowledge and skills. Pre- and post-module tests encourage learners to self-evaluate their baseline learning needs and uptake of taught content.

At the same time evaluation of formative and summative MCQ tests indicates whether desired learning outcomes have been achieved to the developers of learning material. Psychometric analysis of MCQ test results can provide insight into whether questions are well chosen to test learning and of an appropriate level of difficulty and whether they reliably discriminate between good and bad performers [23]. Overall score analysis only provides limited insight though into whether learners knew or guessed an answer correctly or how certain or confident they were of its correctness [24]. Confidence into or certainty of knowledge as well as awareness of uncertainty becomes important when knowledge needs to be applied with immediacy or in potentially high risk situations as is often the case in clinical practice [25, 26]. Reflection on decision making under uncertainty is a significant aspect of clinical reasoning and health professional practice and education [27]. When integrated into formative assessment such reflection can become routine in a learner’s self-evaluation and professional development [28].

One strategy for transferring decision making under uncertainty into the teaching and learning of health professions has been the introduction of certainty (formerly known as confidence) based marking (CBM) of MCQs in formative and summative assessment [29, 30]. In addition to finding the correct answer to a MCQ certainty based marking requires students to state how certain they are that their given answer is correct. Marking schemes have been designed to reward accuracy of answers and honest reporting of degrees of certainty, penalising students for high certainty ratings of incorrect answers and reward acknowledgment of uncertainty while also maintaining reward for correctness of answers [31, 32]. The main argument in support of CBM in summative assessment builds on its ability to distinguish between students who are guessing versus knowing or deducing correct answers.

In formative assessment, CBM of MCQs allows learners and teachers to identify knowledge gaps and gauge certainty of knowledge or reasoning [26]. Evaluation of students’ experience with CBM in formative and summative MCQ tests suggests that it fosters deeper involvement with the tested content, encourages reflection and raises student awareness of areas of uncertainty [33, 34]. The potential for CBM to inform educators as to whether the learning content of their courses achieves intended learning outcomes, for example by increasing learners’ knowledge and skills in combination with increased certainty of knowledge, has been explored as a concept but not been realised in practice [35].

This study aimed to pilot a novel adaptation of CBM in evaluating both e-teaching and e-learning with the use of formative assessment pre-and post-completion of study modules, using certainty ratings of answers to MCQs instead of CBM marking schemes. Success in learning design and delivery would be signified by an overall increase of correct answers in post-tests as well as increased certainty of their correctness compared to pre-tests. Concerns would be raised if correct answers were changed to incorrect or certainty for incorrect answers increased from pre-to post-tests.

## Methods

### Study design

The aims of this pilot study were to investigate the potential utility of formative pre- and post-test MCQs with certainty rating of answers within a virtual learning environment, in terms of:

1. feedback to course designers
2. learner experience

Sets of 10 MCQs were developed for each of five study modules of a one year pharmacotherapeutics course in the PCPP. MCQs were either designed to encourage critical thinking and clinical reasoning by using case scenarios and complex answers from which the most or least appropriate option had to be chosen or they asked about pharmacotherapeutic and clinical knowledge relevant to or contentious in clinical pharmacy practice.

To address the first research question, in addition to answering the MCQs students were asked to rate their certainty of having identified the correct answer for each MCQ on a four point Likert scale. Identical sets of certainty-rated multiple choice questions (CRMCQs) were administered in pre-module tests at the start of each of the five learning modules and post-tests at completion, both available for a limited time period. These covered key aspects of respective learning content while taking care that students weren’t deterred from participation in a voluntary activity by a higher number of questions. Study modules and tests were released approximately monthly over the course year.

The assignment of certainty levels on a four point Likert scale (no idea/ uncertain/ certain/ very certain) was adapted from previous CBM studies which either used three tier Likert scales of low, mid or high certainty or four tier Likert scales expressing certainty in percentages, with ‘very certain’ usually assigned to or understood as high or 80-90 % certainty and’certain’ calibrated in the range of 60–80 % [33, 35, 36]. Students were instructed to choose ‘very certain’ when they felt they were more than 90 % sure their answer was correct and ‘certain’ for less than 90 % certainty. The discrimination between ‘certain’ and very ‘certain’ intended to facilitate observations of differences in certainty levels between pre-and post-tests for those answers where students already had a degree of certainty of correctness in pre-tests.

Automated feedback to students at completion of pre-tests identified which questions they answered correctly or incorrectly. At the same time students also received guidance on which resources and learning materials within the study module would assist them in coming to the correct answer, without explicitly revealing that exact answer. On completion of study modules post-tests were released for a limited time period. Automated feedback now revealed the correct answer and again provided detailed information on where to locate relevant study content, e.g. which lecture, guideline or journal article linked in the module will assist them in finding the answer. Students had the opportunity to revisit their test results if they wanted to check results and answers before repeating tests or for revision before the summative, end of course MCQ test. As test availability was temporally restricted and due to the layout of Blackboard® students had to actively seek out a different section of the course site for this purpose.

To answer the second research question, at the end of the two semester course participants were asked to complete an anonymous online survey (see Additional file 1) answering eleven questions which explored their attitudes towards assigning a certainty rating to their MCQ answers. The survey was based on and adapted from similar instruments investigating the attitudes of students towards CBM and included questions on how CRMCQs affected their approach to learning and module content, using a five point Likert scale (strongly disagree to strongly agree) [30, 34, 37].

#### Data collection and analysis

Overall 1315 matched pairs of answers and certainty ratings between pre- and post-module CRMCQ tests for five modules were downloaded from Blackboard® and CRMCQ data were analysed in Microsoft Excel 2010 and R 3.3.1 [38]. Certainty categories were converted into numerical values, (1 = no idea, 2 = uncertain, 3 = certain and 4 = very certain) and analysed using R. Paired t-tests were conducted to investigate whether certainty levels for correct answers increased between pre- and post- tests for each module. A coding scheme was designed (Table 1) to analyse and describe in more detail any changes of answers given by individual students between pre-and post-tests as well as their assigned certainty ratings. Descriptive statistics were used to analyse survey responses.

**Table 1:** Particpant characteristics

Background variables	No of particpants (%)
Gender	
Female	23 (82)
Male	5 (18)
Years in practice since registration	
2	5 (17.9)
3–5	16 (57.1)
5–10	5 (17.9)
>10	2 (7.1)
Practice setting^a^	
Hospital pharmacy	24 (85.7)
Community Pharmacy	4 (14.3)
Consultant pharmacist in primary care	3 (10.7)
Academia	1 (3.5)
Full-time international students	2 (7.1)

^a^adds to more than 100 % as some pharmacists practiced in multiple settings

## Results

### Analysis of certainty-rated multiple choice questions

Of the 39 students who completed the course, 29 (74 %) provided consent for participation in the study. Not all students who consented to participate completed all pre- and post-module CRMCQs of all five evaluated study modules. Only CRMCQs by students who provided answers to all questions of the pre-and post-tests in a particular study module were evaluated. A median of 25 (23–28) consenting students answered all questions of both pre-and post-tests for the five study modules. Proportionate to course enrolment demographics, 82 % of participating students were female and the majority worked at least part-time as hospital pharmacists with less than 5 years of professional practice. Table 1 describes the participant characteristics.

The overall results reflect favourably on module and learning design. One fifth to one third (21.7–35.2 %) of answers to CRMCQs across the five study modules (M1-M5) were changed from an incorrect to a correct answer between pre- and post-tests (codes 1–3). Students who identified the correct answer to pre-test questions usually also identified it in the post-test (28.0–44.2 %) and the majority increased or didn’t change their certainty of having identified the correct answer in post-tests (codes 4–6). Paired t-tests revealed that an increase in certainty levels of having identified the correct answer in the post-tests was consistent and statistically significant across all study modules (Tables 2 and 3).

**Table 2:** Number of answers changed from incorrect to correct and mean levels of certainty in pre-and post-tests of study modules

Change in certainty answers changed from incorrect to correct in pre- and post tests						
Module	Test	No	Certainty mean	SD	t	*p*-value
1	pre	93	2.07	0.71	−9.29	<0.001
	post	93	3.04	0.79		
2	pre	75	1.97	0.54	−7.78	<0.001
	post	75	2.87	0.74		
3	pre	71	1.8	0.71	−9.64	<0.001
	post	71	2.93	0.72		
4	pre	76	1.87	0.66	−10.8	<0.001
	post	76	3.07	0.77		
5	pre	48	1.96	0.54	−6.54	<0.001
	post	48	2.79	0.68

**Table 3:** Number of correct answers and mean levels of certainty in pre-and post-tests of study modules

Change in certainty for correct answers in pre- and post test						
Module	Test	No	Certainty mean	SD	t	*p*-value
1	pre	92	2.35	0.64	−7.65	<0.001
	post	168	3.14	0.74		
2	pre	134	2.34	0.56	−8.62	<0.001
	post	187	3.05	0.73		
3	pre	124	2.15	0.77	−9.16	<0.001
	post	171	3.1	0.72		
4	pre	116	2.35	0.74	−13.89	<0.001
	post	184	3.66	0.77		
5	pre	112	2.63	1.04	−2.43	0.02
	post	144	3.03	0.75		

Table 4 describes a coding scheme, which in particular assisted in analysing changes in correctness of answers and certainty which occurred with lesser frequency. Percentages of each code assigned for each study module (M1-M5) and the mean across all modules are listed, with numerical values in brackets.

**Table 4:** Coding scheme and changes in answers and certainty between pre-and post-tests for all study modules

Code	Code description	M1 % of code (no)	M2 % of code (no)	M3 % of code (no)	M4 % of code (no)	M5 % of code (no)	Mean %
1	change from IC to CO answer, decreased C^a^	1.2 (3)	1.4 (4)	0.4 (1)	1.3 (3)	1.7 (4)	2.58
2	change from IC to CO answer, unchanged C	8.0 (20)	8.7 (24)	7.5 (18)	7.5 (18)	3.4 (8)	7.02
3	change from IC to CO answer, increased C	26.0 (65)	17.9 (63)	25.0 (60)	25.0 (60)	16.5 (38)	22.08
4	CO answer pre & post, decreased C post	3.2 (8)	1.4 (4)	1.7 (4)	0.8 (2)	1.7 (4)	1.76
5	CO answer pre & post, unchanged C post	5.6 (14)	10.5 (29)	10.4 (25)	14.2 (34)	13.0 (30)	10.74
6	CO answer pre & post, increased C post	19.2 (48)	25.0 (70)	27.1 (65)	29.2 (70)	27.5 (66)	25.60
7	same IC answer pre & post, unchanged C	5.2 (13)	5.8 (16)	2.1 (5)	6.3 (15)	8.3 (19)	5.54
8	same IC answer pre & post, increased C	2.4 (6)	6.4 (18)	2.1 (5)	2.5 (6)	6.9 (14)	4.06
9	same IC answer pre & post, decreased C	2.8 (7)	1.1 (3)	0.4 (1)	0.4 (1)	0.9 (2)	1.12
10	different IC answer, increased C	5.2 (13)	5.0 (14)	7.1 (17)	2.1 (5)	2.6 (6)	4.4
11	different IC answer, unchanged / decreased C	14.4 (36)	7.8 (22)	6.3 (15)	6.3 (15)	10.0 (23)	8.96
12	change from CO to IC answer	6.8 (17)	8.2 (23)	10.0 (24)	4.6 (11)	7.0 (16)	7.32

^a^ *IC* incorrect, *CO* correct, *C* certainty

The same incorrect answer in pre-and post-tests was chosen with a frequency of 4.6–16.1 %, in the majority with unchanged certainty (codes 7–9).

An overall average of 13.4 % of incorrect answers in pre-tests were replaced by a different incorrect answer after completion of study modules in the post-module tests, mostly with decreased certainty (codes 10 and 11). A smaller number of answers (average 7.3 %) were changed from the correct to an incorrect one between pre- and post-tests. Eighty percent of students who chose a correct answer in a pre-test and subsequently changed to an incorrect answer in the post-test (code 12), were either uncertain or had no idea that they had chosen the correct answer in the pre-test. Generally uncertainty was higher when incorrect answers were chosen in a post-test compared to correct answers.

The design of individual MCQs and delivery of study module content was then evaluated more specifically. Module and learning design was reviewed when individual CRMCQ results indicated that study modules may not have offered the learning needed to answer them correctly or MCQs could have been ambiguous or flawed in their design, not testing the actual learning adequately. This was regarded to be the case with high occurrences of a) answers not changed from incorrect to correct, b) increased or high certainty levels attached to incorrect answers in post-tests or c) correct answers in pre-tests changed to incorrect answers in post-tests.

On the other hand, d) a high proportion of correct answers with high certainty ratings in pre-tests would suggest course content was already familiar to or mastered by learners and could be removed or not tested. Applying these parameters to CRMCQ results flagged 14 out of 50 deployed for a review in terms of MCQ formulation and the delivery of examined content, with a) occurring 8 times, b) once, c) once and d) 4 times. This led to changes in the course material and/or MCQs for the following year.

#### Analysis of student survey

Twenty-four of the 29 participants completed an anonymous online survey containing eleven questions relating to their perceptions of benefit and usefulness of assigning a certainty rating to their answers in MCQs in structuring or advancing their learning. The majority of students were positive about their experience with CRMCQs in that they agreed or strongly agreed that assigning a degree of certainty to their answers to MCQs:

- Made them think about how certain they are of the correctness of their answer (92 %)
- Made them aware of what they know and don't know (92 %)
- Assisted in identifying knowledge gaps (83 %)
- Assisted in identifying their guesses (71 %)
- Directed their learning (67 %)
- Was useful for revision (67 %)
- Made them think more carefully about answers (67 %)
- Focused the approach to the topic of study (50 %)
- Made them think more before answering clinical questions in practice (50 %)

Students mostly disagreed or were neutral that CRMCQS:

- Were a waste of time (96 %)
- Limited their approach to the topic of study (79 %)

## Discussion

This pilot study was conducted in a postgraduate clinical pharmacy course and designed to evaluate the addition of a certainty rating of answers to MCQs, exploring whether some of the benefits observed in the application of certainty-based marking in summative testing could be translated into formative assessment [31].

The study investigated the effects of restructuring formative e-assessment of study modules to pre- and post-test CRMCQs with sign-posting to relevant learning resources, with the aim of providing guidance to adult learners in how to structure and prioritise their approach to study in an e-learning environment. Designing MCQs that clearly stipulate expected knowledge and identifying resources for knowledge extension worked well in a post-graduate context where students draw on previous experience. Optimising the instructional system within the VLE for this purpose assisted in meeting expectations of good e-assessment and maximised the utility of feedback for student learning [39–42]. The resulting 21–35 % improvement in correct scores across five study modules aligns with similar findings in other postgraduate health profession programs [43, 44].

The addition of certainty ratings to MCQs in pre-and post-tests for each study module along with the student survey results afforded deeper understanding for course designers whether students improved their knowledge and their ability to apply it. Student survey results indicated that learners regarded answering CRMCQs and feedback in pre-module tests as a guide to study module content. CRMCQs directed their learning, seemingly realising the intent of feeding forward and creating assessment for learning [45]. CRMCQ tests with integrated feedback enabled self-assessment, assisted with revision for the final, summative MCQ test and focused but did not limit students’ approach to the topics of study according to their learning needs [15]. In combination with feedback on incorrectness of answers, which points to obvious gaps in their knowledge, CRMCQs also directed their study efforts to areas of low certainty. The increase in certainty of having chosen the correct answer when that answer was indeed correct was consistent and statistically significant over all study modules.

Student feedback indicates that assigning certainty ratings to MCQs added another stimulus to reflect on their knowledge and learning, raising awareness of their own uncertainty. Students described that they became more conscious of what they know or do not know and seemed to engage more before committing to an answer, which is consistent with previous evaluations of student perceptions of CBM [34, 37].

Certainty of knowledge can be regarded as a surrogate marker for quality and applicability of knowledge in a clinical context. If a learner ‘knows’ the correct answer to a clinical problem but isn’t certain, knowledge will not be readily applied in clinical practice, whereas someone who does have great confidence into an incorrect answer and applies this ‘hazardous knowledge’ may cause inadvertent harm [26]. Interestingly, half the students agreed that as a result of taking CRMCQ tests they think more before answering clinical questions in their practice. Although an association between student reflection on learning and testing with reflective clinical practice hasn’t been established conclusively, this finding could be interpreted as an indicator that CRMCQs enable pharmacists to become more reflective practitioners. Promoting reflection on certainty in learning and practice represents one strategy to engage clinicians in decision making under conditions of uncertainty [27, 46]. It may also assist pharmacists, who at times seem to exhibit a dislike of making decisions under uncertainty, to cognitively resolve apprehension through reflection and conscious awareness [47].

Overall, the combination of pre-and post-module CRMCQ tests resulted in achieving e-design of formative assessment which exhibits many of the hallmarks of good assessment and feedback practice. They seemingly assisted in clarifying goals and standards, promoted self-assessment and reflection, provided feedback and motivation, pointed out strategies how to close knowledge gaps, and as described below, helped to shape teaching [48].

Informing teaching was an integral component of the evaluation design. Utilising certainty ratings with MCQs in pre-and post-tests added an additional gauge for course designers whether MCQs were pitched at an appropriate level or required review. When MCQs were answered correctly by a majority of students in a pre-test it could be concluded that either tested learning content or the question were too basic, leading to revision or removal of either in the future. But when certainty ratings for correct pre-test answers were low, the question would still provide stimulus to learn and engage with study module content, demonstrated by the consistently higher degree of certainty in the post-test compared to pre-tests. The majority of students who identified the correct answer in post-tests increased their certainty between pre-and post-module tests (*p*-values <0.001) which can be regarded as a surrogate marker for deeper learning and understanding [37, 48, 49]. Between 63–78 % of all questions were answered correctly in post-module tests, which may have been expected due to the overall complexity testing and learning content of respective modules.

Changes from a correct to an incorrect answer from pre- to post-test raise potential issues of failure in the delivery of learning content or student engagement. Analysing the certainty ratings assigned to such changes provided some assurance that it is unlikely e-learning design confused or mislead students. Most students were ‘uncertain’ or had ‘no idea’ they had chosen the correct answer in the pre-test which indicates they were making a more or less educated guess at the time. Generally students decreased their certainty on incorrect answers in post-tests compared to pre-tests.

A small number of students gave the same incorrect answer in both tests. In an online environment for postgraduate, clinical education with few opportunities to question students directly as compared to face-to-face or clinical teaching this raises concerns that students may hold on to misconceptions and erroneous or outdated “knowledge”, particularly when certainty increases between pre-to post-tests. Additional undesirable outcomes would be students changing their answer from a correct to an incorrect one or choosing a different wrong answer with increased certainty in a post study module test. All of these outcomes occurred infrequently in this study (≤10 %). As results of both pre- and post-tests did not contribute to overall course marks there may have been little incentive or motivation for students to check their pre-test answers before undergoing the post-test at a later time. The addition of post-test marks to the summative assessment of the course could result in greater motivation to integrate and apply results from pre-tests.

The pilot study results afforded a deeper insight into which study module content was delivered in a manner enabling students to apply it correctly in post-tests. Beyond purely looking at the answers to MCQs in pre- and post-tests certainty ratings provided an enhanced understanding whether content was already known and applied well by students at the beginning of a study module. In addition, high certainty ratings for correct answers in post-tests add certainty for course designers that teaching and learning in a study module have achieved the intended outcomes, and correct guesses are minimally involved in increased correctness of answers. On the other hand, increased certainty in post-tests for incorrect answers given in both pre-and post-tests flags necessary reviews of teaching, MCQ design and strategies for student engagement.

There are a number of limitations to this pilot study which impact on its external validity. Although the participation rate of 75 % in the survey is well above other student surveys the small sample size and predominance of female participants, which was closely related to the enrolment figures, make generalisation of study results difficult. Despite similarity to survey outcomes obtained in comparable settings, knowing the opinion of all students may have provided a more complete picture of students’ experience of CRMCQs. Some of those who didn’t participate in the study may have had disparate views from their peers.

The lack of a control group of students who only answered pre-and post-module MCQs without assigning a certainty rating restricts the validity of the student survey. Although the majority of participants would have had extensive experience with MCQ testing as they completed their undergraduate pharmacy degree in Australia it remains unclear whether the perception of positive impact on learning was generated by the addition of certainty ratings versus just completing MCQs alone. In addition, sign-posting study module content useful in addressing gaps in knowledge on completion of the tests has not been investigated separately from the use of CRMCQs. As the pre-and post-module tests were used formatively some students may not have spent as much effort on identifying correct answers to pre-test MCQs before taking the post-test, particularly as these weren’t linked together, as they may for summative tests. All these factors limit the reliability of results, particularly when considering changes between pre-and post-tests.

Nevertheless, this pilot study adds a new perspective on the usefulness of CRMCQs in formative assessment in an online, postgraduate course where enrolled pharmacists start with varying degrees of knowledge and experience. The results indicate a positive impact on student learning and the potential for evaluating effectiveness of teaching design in achieving the desired learning outcomes, starting to generate proof of the concept suggested by Gardner-Medwin of CBM adding value to formative assessment [34]. In addition the study contributes to the literature on e-learning in pharmacy as well as self and e-assessment [50–52]. Insights based on its findings were used to refine teaching and assessment in the UQ postgraduate pharmacy program to optimise learning for future students.

## Conclusion

Asking students to rate their certainty of correctness of answers to MCQs in formative assessment and providing feedback on how to fill knowledge gaps to increase certainty, creates potential to enhance MCQ testing by encouraging reflection, self-assessment and self-regulation by learners. Students indicated that CRMCQs had a positive impact on their learning by guiding them through online study modules and content, focusing their learning and raising awareness of areas which needed further work or skill development.

The analysis of certainty ratings in addition to the correctness of answers along with trends in changes of answers and certainty between pre- and post-test CRMCQs deployed in an online pharmacotherapeutic, clinical course allowed for more accurate and detailed insights into which topic areas were delivered adequately for students to gain appropriate knowledge and understanding. This pilot study also shows that certainty ratings can assist in identifying topic areas within an online course and MCQ design in pre- and post-module tests that may require adjustment in delivery and design.

## Additional file

Additional file 1: Student questionnaire. (DOCX 11 kb)

## Abbreviations

CBM: Certainty based marking
CRMCQ: Certainty rated multiple choice question
M: Module
MCQ: Multiple choice question
PCPP: Postgraduate clinical pharmacy programs
UQ: The University of Queensland
VLE: Virtual learning environment
