# Supplementary material for: Certainty rating in pre-and post-tests of study modules in an online clinical pharmacy course - A pilot study to evaluate teaching and learning
Source: BMC Med Educ. 2016 Oct 14;16:267. doi: 10.1186/s12909-016-0783-1 (PMC5065079; doi:10.1186/s12909-016-0783-1)
Supplement: Additional file 1: — Student questionnaire. (DOCX 11 kb) [file 12909_2016_783_MOESM1_ESM.docx]

**Additional file 1**

**Student Survey**

| ***Questions*** | ***Answer*** | | | | |
| --- | --- | --- | --- | --- | --- |
| Assigning a degree of certainty to my answers to MCQ in the pre- and post - tests in the study modules: | Strongly agree | Agree | Neither agree/ disagree | Disagree | Strongly disagree |
| Assisted me in identifying knowledge gaps |  |  |  |  |  |
| Directed my learning |  |  |  |  |  |
| Limited my approach to the topic |  |  |  |  |  |
| Made me think about how certain I am of the correctness of my answer |  |  |  |  |  |
| Was a waste of time |  |  |  |  |  |
| Was useful for revision |  |  |  |  |  |
| Made me think more carefully about answers |  |  |  |  |  |
| Assisted me in identifying my guesses |  |  |  |  |  |
| Focused my approach to the topic |  |  |  |  |  |
| Made me aware of what I know and don't know |  |  |  |  |  |
| Made me think more before answering clinical questions in my practice |  |  |  |  |  |
